# Mind the Gap: The Relation Between Identity Gaps and Depression Symptoms in Cultural Adaptation

**DOI:** 10.3389/fpsyg.2020.01156

**Published:** 2020-06-05

**Authors:** Selen Amado, Hannah R. Snyder, Angela Gutchess

**Affiliations:** Department of Psychology, Brandeis University, Waltham, MA, United States

**Keywords:** culture, acculturative stress, depression, identity, self-construal, independence, international students

## Abstract

Personal-enacted identity gaps, defined as the difference between an individual’s self-view and the self that one expresses in communication, affect depression levels of international students living in the United States. Thus, identity gaps are an important construct for understanding the relation between identity and health outcomes. The present research examined the relation between personal-enacted identity gaps formed through communication with Americans and depression, extending previous work by considering the roles of acculturative stress and self-construal and providing one of the first tests of the relation between identity gaps and acculturative stress. A sample of 171 undergraduate and graduate international students studying in the United States was recruited to participate in an online study consisting of self-report questionnaires. Results indicated that as hypothesized, higher levels of personal-enacted identity gaps were associated with depression symptoms and that acculturative stress mediates this relation. However, independent self-construal did not moderate the relation between these variables. Testing additional models revealed multiple ways in which these factors could affect each other. Overall, results suggest that personal-enacted identity gaps, acculturative stress, and depression symptoms are important to consider in tandem in order to understand the emotional experiences of the international students and identify who is at risk for poor mental health. Future longitudinal research is needed to further understand the relations amongst these factors.

## Introduction

The number of international students is rapidly increasing at colleges in the United States. During the past 20 years, numbers have nearly doubled from 450,000 in 1993–1994 to a record high of more than 886,000 in 2013–2014 (Chen et al., 2015). In the 2015–2016 academic year, 1,043,839 international students were enrolled in United States colleges and universities, representing a 7.1% growth from the previous year (Institute of International Education, 2016). This number is expected to continue to grow; thus, research is needed on the factors contributing to the cultural adjustment of these students and their well-being. International students are a high-risk population who are vulnerable to stress due to the process of adapting to a new country. This stress can have negative psychological outcomes such as anxiety and depression.

Acculturative stress is the psychological impact of adaptation to a new culture (Smart and Smart, 1995). Thus far, research show that English fluency, social support satisfaction and social connectedness are all predictors of acculturative stress in international students (Yeh and Inose, 2003), and that acculturative stress is positively associated with depression symptoms (Wei et al., 2007). The impact of internal factors, psychological factors related to the individual’s attributes, traits, emotions, thoughts, personality etc., on acculturative stress needs more exploration in the field (Miranda and Matheny, 2000). For example, self-concept is an internal factor that can be associated with negative outcomes when it is out of sync with expression of the self. These identity inconsistencies are called *identity gaps* and have been found to be related to depression symptoms among international students at United States colleges (Jung and Hecht, 2004). The personal-enacted identity gap, which is defined as the difference between individuals’ self-view and the self they express in communication, significantly predicts depression in international students (Jung et al., 2007). Studies on acculturative stress models in international students have focused on language, educational stressors, sociocultural stressors, discrimination, and practical stressors as predictors of acculturative stress (Smith and Khawaja, 2011); no previous studies have focused on identity gaps as a factor predicting acculturative stress. The present study aims to explore whether there is a positive association between personal-enacted identity gaps and acculturative stress. Furthermore, the study extends the literature by examining the role of acculturative stress as a possible mediator of the relation between personal-enacted identity gaps and depression symptoms in international students.

Self-construal refers to the ways in which people think about and conceptualize the self. Across cultures, there is variation in the degree to which the self is thought of as independent from the context, or conceptualized in relation to others (Markus and Kitayama, 1991). Viewing the self as separate from others reflects an independent self-construal, in which behavior is organized and made meaningful primarily by reference to one’s own internal thoughts, feelings, and actions, rather than by reference to the thoughts, feelings, and actions of others (Markus and Kitayama, 1991). This variation in the view of the self has an effect on cross-cultural adaptation in that international students with an independent self-construal show lower levels of perceived stress during cross-cultural adaptation to the United States (Cross, 1995). This is in line with suggestions that an independent self-construal is related to better psychological and sociocultural adjustment to Western cultures (Oguri and Gudykunst, 2002). The current study aims to extend these findings by investigating whether self-construal moderates the relation between personal-enacted identity gaps and acculturative stress, and the relation between acculturative stress and depression.

Thus, the main purpose of this study is to examine the relation between personal-enacted identity gaps, acculturative stress and depression symptoms in international students studying in the United States, to explore the role of acculturative stress as a mediator in this relation, and to test the moderating effect of self-construal.

### Identity Gaps

International students studying in the United States may experience discontinuities between their self-concepts and how they are seen by Americans (Jung et al., 2007). These inconsistencies, that theoretically may arise from cultural differences and communication barriers, are called ‘identity gaps.’ There are four aspects of identity recognized by The Communication Theory of Identity: personal, relational, enacted, and communal layers (Hecht, 1993). One that has emerged to be particularly important for international students’ well-being is the personal-enacted identity gap, defined as the difference between an individual’s self-view and the self expressed or performed in communication (Jung and Hecht, 2004). We use the phrase ‘self expressed’ throughout the manuscript to refer to the self enacted in communication as perceived by the individual, not by others. For example, one can see oneself as open minded but, in certain situations, avoid discussion with people of differing opinions (Jung and Hecht, 2004). The identity that is enacted, one who is unwilling to engage in discussion, is different from the self-image of one who is open-minded. Similarly, the communicator can present oneself in line with a social or a communication partner’s expectations in order to maintain the relationship, at the expense of expressing their inner thoughts (Jack, 1991).

#### Personal-Enacted Identity Gaps and Depression

Research with international students shows that personal-enacted identity gaps can be strongly related to depression level. Personal-enacted identity gaps formed in communication with Americans predicted elevated levels of depression in Korean immigrants as well as international students (Jung et al., 2007; Jung and Hecht, 2008). This effect may be explained by the motive to present the self as one sees oneself. International students’ difficulty expressing themselves in accordance with their self-view to American communication partners may lead to frustration, especially when the students have moved from another country to deliberately start a new life with this population. This may create the feeling that they are disadvantaged in social situations with Americans. Identity gaps formed in communication with Americans may predict elevated levels of depression in international students. The first hypothesis of this study was a replication of prior studies:

**H1:** Personal-enacted identity gaps formed by communication with American acquaintances is associated with depression symptoms in international students.

### Acculturative Stress

Adaptation to a new country and culture is stressful (Sandhu and Asrabadi, 1994). Acculturative stress is the psychological impact of adaptation to a new culture (Smart and Smart, 1995), and is recognized by acculturation researchers (Berry, 1970) as an important way of conceptualizing psychological difficulties in the adaptation process (Wang and Xiao, 2014). Acculturative stress is defined as “a response by people to life events that are rooted in intercultural contact” (Berry, 2006, p. 43) and is influenced by multiple factors (Berry, 1990) including language fluency, social support satisfaction, and social connectedness (Yeh and Inose, 2003). Miranda and Matheny (2000) emphasized the need to consider internal factors that predict acculturative stress rather than external factors such as demographics. The present study focuses on internal factors by investigating the relation between personal-enacted identity gaps and acculturative stress in international students. A systematic review of 64 studies that examined predictors of psychosocial adjustment of international undergraduate and graduate students in the United States suggests that identity gaps are a micro-level factor deserving attention in the psychological adjustment literature (Zhang and Goodson, 2011). Previous research has not examined identity gaps as a predictor of acculturative stress; the present study aims to look at this relation in order to identify some sources of international students’ psychological stress. Therefore, the second hypothesis of the study was:

**H2:** The personal-enacted identity gaps are positively associated with acculturative stress in international students.

#### Acculturative Stress and Depression

International students are a vulnerable population who are faced with a new set of basic values and beliefs and so are continually challenged to accommodate themselves to a variety of cultural differences in the host country (Mori, 2000). The stress related to adaptation to a new culture is associated with negative emotional states such as anxiety and depression (Williams and Berry, 1991). A study by Han et al. (2013) found that self-reported symptoms of depression and anxiety rates among international graduate students in a university in the United States (45% and 29%, respectively) were higher (14% and 9%, respectively) than domestic students. Acculturative stress in international students is associated with these negative outcomes, with higher levels of acculturative stress associated with more depression symptoms (Jackson et al., 2013). The associations between acculturative stress and mental health outcomes have been identified in East Asian (i.e., Chinese and Korean) international students for depression symptoms (Wei et al., 2007; Rice et al., 2012; Hamamura and Laird, 2014; Lee and Ciftci, 2014) and in Mexican American college students for anxiety and depression symptoms (Crockett et al., 2007). Consistent with these findings, the third hypothesis of the study was a replication of previous studies:

**H3:** Acculturative stress is positively associated with depression symptoms in international students.

#### Acculturative Stress, Personal-Enacted Identity Gaps, and Depression Symptoms

We predicted that the positive relation between personal-enacted identity gaps and depression symptoms operates via acculturative stress; international students’ feelings of not being able to express themselves in accordance with their self view in communication with Americans may be a possible acculturative stressor that contributes to feelings of depression. Therefore, the fourth hypothesis of the study was:

**H4:** Acculturative stress mediates the relation between personal-enacted identity gaps and depression symptoms.

### Self-Construal

Self-construals are conceptualized as constellations of thoughts, feelings, and actions concerning an individual’s relation to others and the self as distinct from others (Singelis, 1994). Culture can influence the ways in which individuals define themselves in relation to their social context (Markus and Kitayama, 1991). What individuals believe about the relation between the self and others and the degree to which they see themselves as separate from or connected with others reflects their self-construal.

An independent self-construal involves a conception of the self as an autonomous, independent person. The self is assumed to be a complete, whole, autonomous entity, without other people (Markus and Kitayama, 1991), referring to their own abilities, attributes, characteristics and goals rather than referring to the thoughts, feelings or actions of others (Singelis, 1994). In contrast, an interdependent self-construal is defined as a “flexible, variable” self that emphasizes external public features such as status, roles and relationships, belonging and fitting in, occupying one’s proper place, engaging in appropriate action, and being indirect in communication (Singelis, 1994). Western cultures tend to be associated with more independent self-construals whereas Eastern cultures tend to be associated with more interdependent self-construals (Markus and Kitayama, 1991).

Independent self-construal is a complex construct that may be of importance when examining the relation between personal enacted identity gaps and acculturative stress. Previous literature shows that this construct is associated with biases in how people interpret information about the self (Lee et al., 2000). It is possible that a dominant independent self-construal may also be associated with the extent to which others influence the self. On the other hand, a dominant independent self-construal may be associated with emphasis on self-consistency. These possible effects of self-construal may be associated with the extent of acculturative stress experienced related to expressing the self in accordance with self-view. Therefore, this study investigated the moderating role of independent self-construals on the association between acculturative stress and personal enacted identity gaps. The fifth hypothesis of this study was:

**H5:** The independent self-construal is a moderator between personal-enacted identity gaps and acculturative stress.

Research shows that this cultural aspect of the self is related to psychological adjustment. A study on the influence of self-construals on psychological and sociocultural adjustment of temporary residents found that independent self-construal predicted psychological and sociocultural adjustment whereas interdependent self-construal was not related to psychological adjustment (Oguri and Gudykunst, 2002). This may be explained by the cultural-fit between the dominant self-construal of the host country, United States, and the dominant self-construal of the international student (Oguri and Gudykunst, 2002). When an individual’s self-construal fit the host country’s dominant independent self-construal, the student was better adjusted. Another perspective suggests that having an independent self-construal itself, regardless of the match with the host society, is conducive to better cross-cultural adaptation. This perspective is based on a study that found that participants with higher independent self-construals showed better psychological adjustment regardless of the similarity between their self-construal and the mean score of the host sample (Yang et al., 2006), a pattern that goes against the cultural-fit hypothesis. This suggests that self-construals, especially the independent self-construal, have a strong influence on coping, psychological and sociocultural adjustment, and thus may affect depression symptoms. The present study measures both independent and interdependent self-construal but focuses on the moderation effect of independent self-construal on acculturative stress and depression symptoms. Therefore, the final hypothesis of the study was:

**H6:** The positive effect of having an independent self-construal will moderate the relation between acculturative stress and depression symptoms.

In conclusion, the main purpose of the study is to test the association between international students’ personal-enacted identity gaps, acculturative stress and depression symptoms. We predicted that acculturative stress would be a mediator in this relation, and that there would be a moderating effect of self-construal. As the current study used a correlational design, this model serves as an initial test of the relations amongst these variables and we cannot claim causal relations.

## Materials and Methods

### Participants and Procedure

The participants of the study were 171 international students studying in the United States. Three additional participants who stated that they were born in the United States were removed because they did not meet the eligibility criteria. Our target was to recruit at least 30 participants per path, for the five paths in the hypothesized model, for a total of at least 150 participants. The data were not analyzed until the complete sample had been collected. We conducted a power analysis using Monte Carlo Power Analysis for Indirect Effects software (Schoemann et al., 2017), with effect sizes estimated from previous studies reporting correlations between acculturative stress and depression symptoms (Oh et al., 2002; Crockett et al., 2007; Wei et al., 2007; Rice et al., 2012; Jackson et al., 2013; Hamamura and Laird, 2014; Huang and Mussap, 2018), personal-enacted identity gap and depression symptoms (Jung et al., 2007; 2008; Jung, 2013), and personal-enacted identity gap and the perceived discrimination subscale of the acculturative stress measure (no studies reported correlations with the full acculturative stress scale; Jung et al., 2007; Wadsworth et al., 2008). Given the sample size of 171, power to detect the indirect effect was virtually 1. The final sample consisted of 112 female and 59 male students. The students were undergraduate (64%) and graduate students (36%) enrolled in a higher education institution. Eligible participants were living in the United States for less than four years and English was not their first language. International students from English speaking countries (e.g., Canada) did not meet inclusion criteria for the study. Participants were from 39 different countries, with the most participants from China (46%) and India (14%) (other countries < 10% each, see Supplementary Table S1) and reported a variety of native languages (48.5% Chinese, 10% Turkish; remainder were < 10%). The participants had been enrolled in a higher education institution in United States for a mean of 20 months (range 0–48 months) and the age range of the participants was 18–35 with a mean of 22.27 (SD = 3.8, range 18–35). The 5-point self-report scale of English fluency revealed that the percentage of participants who reported their fluency level as 5 corresponding to fluent and proficient was 46%, participants who reported their fluency level as a 4 corresponding to functional fluency level was 43%, and the rest of the participants reported a lower level on the 5-point scale ranging from none to limited.

This study was carried out in accordance with the recommendations of the Common Rule, Department of Health and Human Services (HHS) through the Office for Human Research Protections (OHRP). The protocol was approved by the Brandeis University Institutional Review Board. All subjects gave written informed consent in accordance with the Declaration of Helsinki.

Participants were recruited through various methods. International students studying at Brandeis University were recruited through the Psychology department research participant pool, campus flyers, and an email sent by the Brandeis International Students and Scholars Office. Participants from other colleges (listed in Supplementary Table S2) were recruited through postings on Facebook pages and groups associated with international student groups. After participants clicked on the link provided, they started the study with eligibility questions. For those who were eligible, they next completed the consent form to start the survey, followed by questionnaires measuring acculturative stress, personal-enacted identity gaps, depression symptoms, and self-construal (participants also completed the Perceived Stress Scale, not analyzed here). Questionnaires were presented in random order to avoid any order effects. Finally, the demographics questionnaire was presented at the end of the survey. Questions were presented one at a time on the screen to ensure that participants did not miss any questions. After completing the online Qualtrics survey (20–45 min), the participants recruited through Brandeis courses received credit toward the course requirements for their participation. All other participants received a one-time use $3 Amazon gift card as reimbursement. The study was approved by the Brandeis University Institutional Review Board.

### Measures

#### Personal-Enacted Identity Gap Scale (Jung and Hecht, 2004)

The scale consists of 11 items (α = 0.89) scored on a 7-point Likert-type scale ranging from 1 = strongly disagree to 7 = strongly agree. The possible range of scores is 11–77. The scale was modified by replacing ‘my communication partners’ with ‘American acquaintances’ in each item. Sample items include: “When communicating with my American acquaintances I often lose sense of who I am.” and “I freely express the real me in communication with my American acquaintances.”

#### Acculturative Stress Scale for International Students (ASSIS; Sandhu and Asrabadi, 1994)

The scale consists of 36-items (α = 0.93) rated on a 5-point Likert scale, ranging from strongly disagree to strongly agree. The ASSIS consists of seven factors: Perceived Discrimination (8 items), Homesickness (4 items), Perceived Hate (5 items), Fear (4 items), Stress Due to Change/Culture Shock (3 items), Guilt (2 items), and Non-specific Concerns (10 items). Scores could range from 36 to 180. Sample questions include: “I feel rejected when people are sarcastic toward my cultural values” and “Others are biased toward me.”

#### Center for Epidemiologic Studies-Depression Scale (CES-D; Radloff, 1977)

The CES-D is a self-report scale consisting of 20 items (α = 0.90) that was developed to assess current levels of depression symptoms. The items were rated on a 4-point scale [1- Rarely or None of the Time (Less than 1 Day), 2- Some or a Little of the Time (1–2 Days), 3- Occasionally or a Moderate Amount of Time (3–4 Days), 4- Most or All of the Time (5–7 Days)]. Total scores can range from 0 to 60. Sample questions include: “During the past week, I thought my life had been a failure” and “During the past week, I felt that people dislike me.”

#### Self-Construal Scale (Singelis, 1994)

The Self-Construal Scale consists of 30 items with 15 items (α = 0.82) in the independent subscale and 15 items (α = 0.77) in the interdependent subscale. The scale was rated on a 7-point Likert scale ranging from 1 = strongly disagree to 7 = strongly agree. A separate score is obtained for each subscale, with a possible range from 15 to 105. Sample items include “I have respect for the authority figures with whom I interact” and “My personal identity, independent of others, is very important to me.”

#### Demographics

We obtained information on demographic characteristics with an 11-item questionnaire. The items asked for the participant’s age, gender, country of birth, number of years lived in home country, country of residence, number of years lived in the United States, number of years enrolled in a higher education institution in the United States, educational status: undergraduate or graduate, native language, English fluency level (*very limited to fluent and proficient*), and race.

#### Analytic Approach

In our analysis, we first assessed the mediation model, testing (1) the total effect of personal-enacted identity gaps (X) on depression symptoms (Y) and (2) the mediation effect of acculturative stress (M). Next, we tested a moderated mediation model, where the independent self-construal (W) acts as a moderator for personal-enacted identity gaps and acculturative stress within the mediation model. Finally, because the study was cross-sectional, precluding conclusions about the causal ordering of variables, we tested alternative mediation models with the order of variables rotated, and an alternative multiple regression model. Age and gender were included as covariates in all analyses given established age and gender differences in depression (e.g., Nolen-Hoeksema, 2001; Kuehner, 2003; Kessler et al., 2012), with some evidence that gender differences extend across cultures (e.g., to Hispanic immigrants; Allen et al., 1998). All variables were mean centered for use in interaction terms. For all scales, scores that were outliers, defined as ±3 SDs from the mean, were excluded, resulting in removal of five scores from analysis. Analyses were run in Mplus, using full information maximum likelihood (FILM) to handle missing data and bias-corrected 95% confidence intervals (10,000 repetitions) (Muthén and Muthén, 2012).

## Results

### Introduction

Means and standard deviations for the measures, and the correlations amongst them, are presented in Table 1.

**TABLE 1:** Means, standard deviations and correlations of variables.

**Variable**	**ACS**	**Dep**	**PEI**	**Indep**	**Age**	***M***	***SD***
Acculturative stress (ACS)						98.48	22.74
Depression symptoms (Dep)	0.39**					15.22	9.82
Personal-enacted identity gaps (PEI)	0.33**	0.35**				39.71	11.03
Independent self-construal (Ind)	−0.21*	−0.26*	−0.33**			73.92	10.45
Years in United States	0.038	–0.07	–0.010	0.21*	0.20*	1.80	1.22

**p < 0.001; *p < 0.01.

### Hypothesis Tests

**FIGURE 1:**
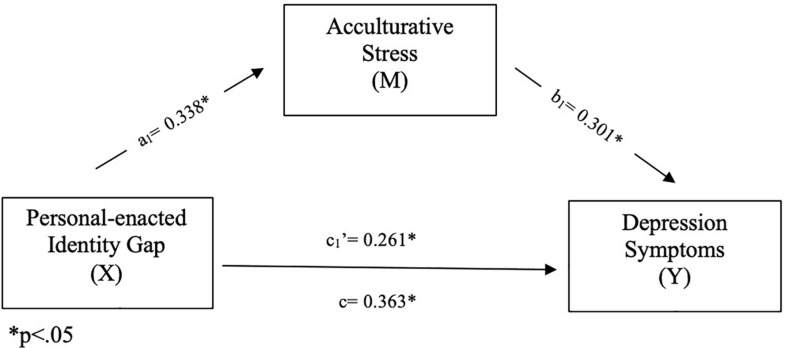
Mediation model. In this model, personal-enacted identity gaps were positively associated with acculturative stress in international students and acculturative stress was positively associated with depression of personal-enacted identity gaps on depression symptoms though the direct effect of personal-enacted identity gaps on depression symptoms remained significant. ^∗^*p* < 0.05.

The results of the complete model are presented in Table 2 and Figure 1.

**TABLE 2:** Results for the total effects model and the hypothesized mediation model: personal-enacted identity gaps (X) and depression symptoms (Y) mediated by acculturative stress (M).

**Outcome variable**	**Predictor**	***b***	**SE**	***b/SE***	**β**	***p***	***R*^2^**
**Total effects model:**							
Dep	PEI	0.315	0.061	5.159	0.360	<0.001	0.139
	Age	0.291	0.170	1.705	0.116	0.088	
	Gender	−0.487	1.527	−0.0.319	−0.050	0.750	
**Mediation model:**							
ACS	PEI	0.688	0.152	4.537	0.338	0.001*	0.126
	Age	0.779	0.464	1.679	0.132	0.093	
	Gender	2.378	3.582	0.64	0.106	0.507	
Dep	PEI	0.228	0.061	3.725	0.261	0.001*	0.219
	ACS	0.129	0.033	3.928	0.301	<0.001*	
	Age	0.190	0.165	1.153	0.075	0.249	
	Gender	−0.774	1.509	−0.513	−0.080	0.608	

	Total, indirect and direct effects						

PEI – Dep total		0.317	0.061	5.160	0.363	<0.001*	
PEI – Dep indirect effect		0.089	0.030	2.929	0.102	0.003*	
Direct effect of PEI		0.228	0.061	3.725	0.261	<0.001*	

*p < 0.05.

#### Total Effect Model

The first hypothesis was that personal-enacted identity gaps formed by communication with American acquaintances are associated with depression symptoms in international students. In the total effects model, personal-enacted identity gaps were significantly associated with depression symptoms (c: β = 0.360, *p* < 0.001) supporting hypothesis one.

#### Mediation Model

Personal-enacted identity gaps were positively associated with acculturative stress in international students, supporting hypothesis two (a: β = 0.338, *p* < 0.001). Acculturative stress was in turn positively associated with depression symptoms, supporting hypothesis three (b: β = 0.301, *p* = 0.003). The effects of personal-enacted identity gaps on depression symptoms were significantly mediated by acculturative stress (Indirect effect: β = 0.102, *p* = 0.003, 95% bias-correct bootstrapped confidence interval 0.039–0.162), supporting the fourth hypothesis of the study. The direct effect of personal-enacted identity gaps on depression symptoms remained significant (c’: β = 0.261, *p* < 0.001). The percent of the total effect mediated was 28.1% (see Table 2). There were no significant effects of the covariates age and gender.

#### Moderated Mediation Model

We had predicted that a higher independent self-construal would weaken the relation between the personal-enacted identity gaps and acculturative stress, but the effect was not significant (w: β = −0.116, *p* = 0.222), thus failing to support hypothesis five. In addition, independent self-construal was examined as a moderator of the association between acculturative stress and depression symptoms within the mediation model. The effect was not significant, (w: β = −0.039, *p* = 0.532), thus failing to support hypothesis six (see Table 3). To summarize, controlling for age and gender, acculturative stress was a significant mediator for personal-enacted identity gaps and depression symptoms but independent self-construal did not significantly moderate this mediation effect (see Table 3). There were no significant effects of the covariates age and gender.

**TABLE 3:** Results for the hypothesized moderated mediation model: personal-enacted identity gaps (X) and depression symptoms (Y) mediated by acculturative stress (M), and moderated by independent self-construal (W).

**Outcome variable**	**Predictor**	***b***	**SE**	***b/SE***	**β**	***p***	***R*^2^**
ACS	PEI	0.610	0.164	3.714	0.300	0.001*	0.145
	Ind	–0.181	0.195	–0.0.930	–0.082	0.352	
	Ind × PEI	–0.023	0.019	–1.222	–0.116	0.222	
	Age	0.790	0.487	1.622	0.134	0.105	
	Gender	1.623	3.751	0.433	0.072	0.665	
Dep	PEI	0.188	0.067	2.822	0.215	0.005*	0.248
	ACS	0.129	0.034	3.766	0.300	< 0.001*	
	Ind	–0.135	0.064	–2.108	–0.142	0.035*	
	Ind × ACS	–0.001	0.002	–0.625	–0.039	0.532	
	Age	0.190	0.171	1.110	0.075	0.267	
	Gender	–0.848	1.518	–0.559	–0.087	0.576	

	Total, indirect and direct effects						

PEI – Dep total		0.266	0.069	3.838	0.305	< 0.001*	
PEI – Dep indirect effect		0.078	0.031	2.533	0.090	0.011*	
Direct effect of PEI		0.188	0.067	2.822	0.215	0.005*	

*p < 0.05.

### Evaluation of Other Models

We next tested alternative models to assess whether our hypothesized model best captured the relations amongst variables. The alternative models and results are available in Supplementary Material (Supplementary Figures S1–S5 and Supplementary Tables S3–S7). Results suggest that our model is not unique in accounting for relations amongst the variables; based on Bayesian Information Criterion (BIC) and Akaike Information Criterion (AIC) comparisons, some rotations of the models were as good, or better, at accounting for the relations amongst variables (see Supplementary Table S8). We suggest that these patterns of results highlight the importance of studying bidirectional relations among these variables in a longitudinal design in order to understand mental health and risk factors for international students.

### Regression Analysis

Because multiple models were significant and adequately accounted for effects, we adopted an alternate approach to analyses. Regression analyses allow us to move away from making directional claims about the relations amongst variables. The initial model tested the effects of personal-enacted identity gaps, acculturative stress, and independence on depression symptoms, with age and gender as covariates. The model also included the interaction terms (personal-enacted identity gaps × acculturative stress; independence × personal-enacted identity gaps; independence × acculturative stress). Because none of the interactions approached significance (*p*s > 0.20), these were removed from the final model. The results of the model, *R*^2^ = 0.24, are presented in Table 4. Of note, all three factors (personal-enacted identity gaps, acculturative stress, and independence) are associated with depression symptoms, even when controlling for the others. Thus, the results further underscore the potential for personal-enacted identity gaps and acculturative stress to serve as risk factors for poor mental health in international students, whereas independence self-construal may play a protective role.

**TABLE 4:** Results for the regression model.^†^

**Outcome variable**	**Predictor**	***b***	**SE**	***b/SE***	**β**	***p***	***R*^2^**
Dep	PEI	0.194	0.064	3.022	0.223	0.003*	0.238
	ACS	0.125	0.034	3.711	0.293	< 0.001*	
	Ind	–0.134	0.062	–2.146	–0.140	0.032*	
	Age	0.196	0.169	1.158	0.078	0.247	
	Gender	–0.817	1.534	–0.533	–0.084	0.594	

^†^In an exploratory analysis, we added years lived in the United States to the regression model. This variable did not play a large, or significant, role, with a standardized coefficient beta = −0.068, *p* = 0.357; VIF = 1.12. *p < 0.05.

Supplementary Material include results for *post hoc* analyses that were conducted to assess potential contributions of additional variables that might account for, or otherwise alter, potential relations amongst the variables of interest. We conducted exploratory analyses with the following factors: English fluency, years in the United States, cultural similarity. Supplementary Material also include results on whether effects held for a subsample consisting of Asian participants.

## Discussion

### Association Between Personal-Enacted Identity Gaps, Depression Symptoms, and Acculturative Stress

This study was the first to examine the relations amongst personal-enacted identity gaps, acculturative stress and depression symptoms within international students in the United States in a cross-sectional model. As hypothesized, we found that personal-enacted identity gaps were significantly associated with depression and acculturative stress in this population. The association between personal-enacted identity gaps and depression symptoms is consistent with prior evidence (Jung et al., 2007; Jung and Hecht, 2008), offering novel evidence linking identity gaps to depression with consideration of mediating factors, such as acculturative stress. The mechanisms linking identity gaps to depression are unknown. We speculate that this difference between an individual’s self-view and self expressed through communication may negatively impact the relationships and social standing of the student and create loneliness. International students’ difficulty representing themselves accurately to Americans may contribute to frustration, especially when the students have moved from another country to deliberately start a new life with Americans. This may create the feeling that they are disadvantaged in social situations with Americans. It may also make the students feel that they have two separate identities: their self-view, and their identity when speaking in English to Americans. All these possible negative effects of personal-enacted identity gaps in international students may be associated with depression symptoms.

Previous research has not assessed the relation between personal-enacted identity gaps and acculturative stress, despite a review suggesting that identity gaps are a micro-level factor deserving attention in the psychological adjustment literature (Zhang and Goodson, 2011). Our findings showed that higher levels of personal-enacted identity gaps are associated with higher levels of acculturative stress in this population. The way in which identity gaps are linked to acculturative stress is unknown. It may be that not being able to show their perceived self to Americans make the students feel like they will be judged and discriminated against because of their inability to be themselves. Identity gaps may be associated with individuals’ fear that they will not be able to show their perceived selves to Americans throughout their time in the United States or feelings of guilt related to thinking that they are tricking Americans by not expressing their self-viewed identity. To assess which factors related to identity gaps are associated with acculturative stress, future studies can test the effects of personal-enacted identity gaps on specific factors (e.g., Guilt; Homesickness) of the Acculturative Stress Scale.

Our findings also support the idea that acculturative stress is associated with depression symptoms in international students, consistent with previous studies (e.g., Lee et al., 2004; Wei et al., 2007; Rice et al., 2012; Jackson et al., 2013; Hamamura and Laird, 2014; Huang and Mussap, 2018). In addition, previous studies have found that higher levels of acculturative stress are also associated with higher suicidal ideation (Taliaferro et al., 2019). Future studies should examine the relation between acculturative stress, depression and suicidal ideation in this population.

Although our study reveals some evidence for relations between the predicted factors, other factors may contribute. *Post hoc* analyses (see Supplementary Material) indicate that English fluency significantly predicted acculturative stress and depressive symptoms in the current sample of international students. This finding suggests that international students with poor English fluency may be particularly at risk of heightened levels of acculturative stress and depressive symptoms, and thus may be an important consideration for interventions focused on language. For example, conversation workshops and English proficiency orientations may help international students feel more comfortable in their linguistic interactions with American peers.

### Mediation Effects of Acculturative Stress

In our hypothesized model, we predicted that acculturative stress would mediate the relation between personal-enacted identity gaps and depression symptoms. Our hypothesis was supported; acculturative stress mediates ∼28% of the effect of personal-enacted identity gaps on depression symptoms. Our findings could suggest that when international students feel a difference between their self-view and self expressed in communication with Americans, these feelings may be related to stress about their fit and adaptation to American culture, contributing to symptoms of depression.

Our data also indicate that multiple relations amongst factors could contribute to depression symptoms. Because the current study was cross-sectional, we cannot draw conclusions about the directionality of effects, and tested additional mediation models to explore alternative orderings of the relations between acculturative stress, depression symptoms and personal-enacted identity gaps also fit the data. Some models were as good or better at explaining the relation amongst these variables (see Supplementary Figures S1–S5), and future longitudinal studies should investigate temporal precedence to test for causal relations. Specifically, a rotation in which depression symptoms mediate the relation between acculturative stress and personal-enacted identity gaps had a better fit (rotation 2, Supplementary Figure S3). Future longitudinal studies should investigate whether personal-enacted identity gaps can be an outcome of changes in interpersonal communication style related to depression symptoms such as excessive reassurance-seeking, or changes in self-perception associated with depression such as self-doubt and low self-regard (e.g., Joiner Alfano and Metalsky, 1993; Weinstock and Whisman, 2007). The change in the way depressed individuals communicate with others as a result of depression symptoms may make them feel as if they are not enacting their usual self and thus cause personal-enacted identity gaps.

Another rotation in which personal enacted identity gaps mediate the relation between acculturative stress and depression symptoms also had a better fit (rotation 3, Supplementary Figure S4). This finding may be related to Jung et al. (2007) finding that personal-enacted identity gap is a significant mediator between acculturation (which is negatively correlated with acculturative stress; Oh et al., 2002) and depression symptoms. It may be that acculturative stress and personal-enacted identity gaps are bidirectionally related future longitudinal research is needed to understand the directionality of the relation. Given lack of certainty regarding the causal direction of these effects, interventions for prevention and reduction of depressive symptoms in international students may be most effective when they target both acculturative stress and personal-enacted identity gaps and adopt a holistic approach to integrate these factors. In addition, it is possible that other mechanisms not assessed in this study, such as personality traits or other factors that cause individuals to self-select to study internationally, contribute to the multiple types of relations revealed in this study.

### Moderation Effects of Independent Self-Construal

Based on findings that higher levels of independent self-construal are related to better psychological and sociocultural adjustment in international students (Oguri and Gudykunst, 2002; Yang et al., 2006; Wang, 2009), we had hypothesized that students who have a higher independent self-construal would be able to cope with the effects of acculturative stress better through engagement in better coping strategies and so the effect of acculturative stress on depression symptoms would be lower. Contrary to our expectations, independent self-construal did not moderate the effects of personal-enacted identity gaps, failing to buffer the effects of the level of stress related to adapting to a new culture.

### Limitations and Future Studies

In addition to the alternative models and potential factors highlighted in the previous section, this study had a number of limitations. First, a sample of this size cannot capture all of the breadth of the entire group of international students in the United States. The data were collected entirely from colleges in the East Coast, with 60% of the sample from Brandeis University. According to statistics from the Institute of International Education, California has the largest international student population in the United States, but the state was not represented in our sample. In addition, our sample did not match the distribution of other countries reflecting in the international student population: Chinese students are overrepresented in our sample (44% compared to the distribution in the United States of 31%), whereas other nations are underrepresented. The second limitation of this study is that it is cross-sectional and relies on self-report, and thus we are not able to make causal claims. Although most prior studies with international students (e.g., Zhang and Goodson, 2011; Li et al., 2019) share this limitation of being cross-sectional, longitudinal studies are needed to more clearly understand the precedence and patterns of these effects over time. Our examination of alternate models (see the section “Conclusion”) suggests that many potential relations amongst variables are possible, and it is likely that many effects are bidirectional (e.g., depression leading to perception of greater identity gaps and acculturative stress). Although this study contributes to the literature by examining the relations amongst a unique group of variables, longitudinal data would help to further distinguish the relations amongst different factors and to identify which model best reflects these relations.

In addition, the ways in which the concept of identity gaps relates to concepts such as self-consistency, expression of personality, or expression of emotions is unclear. The personal-enacted identity gap questionnaire assesses the extent to which an individual feels as if there are gaps between her expressed self and her self-view in a communication context rather than comparing differences in one’s actual and ideal self-states (i.e., self-discrepancy theory; Higgins, 1987). Future studies directly comparing measures of these constructs and including these factors in a single study will help to address questions about the interrelatedness of these concepts, and further refine which mechanisms contribute the most to depressive symptoms in international students. In addition, examining these constructs across a range of cultural environments is important to establish the validity of these very constructs. There are multiple studies that have been conducted in non-WEIRD (Western, Educated, Industrial, Rich, or Democratic; Henrich et al., 2010) populations that show the psychological costs of having low consistency and feeling unable to openly express one’s self-attributes. These studies were conducted with immigrant groups (Jung and Hecht, 2008; Urban and Orbe, 2010) and international students (Jung et al., 2007; Zhang and Goodson, 2011). Despite this research in a variety of populations, the model may make some assumptions about the underlying nature of the self in line with Western views of the self. That is, Easterners may think of the self in a highly context-dependent manner, whereas Westerners may think of the self as a more absolute and stable entity (Oyserman and Lee, 2008; Chiao et al., 2010). Although the model considers context, with a focus on communication contexts, in how one thinks of the self, some of the items imply an absolute or “real” self that may not be compatible with the context-dependent self of Easterners. Further, the emphasis that the construct of identity gaps places on the open expression of one’s self and consistency with inner thoughts and desires, may reflect a Western bias for sharing and communicating about the self (Markus and Kitayama, 1991). Furthermore, the reliance of this work on self-report methods should be validated with assessment of real-world outcomes (e.g., clinical research addressing how the self is perceived in therapy, and the effectiveness of different approaches).

### Future Implications

The findings of this study could inform ways to reduce international students’ depression levels. It is possible that addressing personal-enacted identity gaps may reduce stress, and thus reduce levels of depression. However, at this point it is premature to target personal-enacted identity gaps, as reducing acculturative stress or improving language fluency may play larger roles in reducing depression symptoms. Should future research indicate a causal role for identity gaps in increasing acculturative stress, colleges in the United States could aim to reduce depression in international students by targeting the personal-enacted identity gap through interventions and training on communication with American acquaintances. Furthermore, during the school year, students with a high risk for depression may be able to be identified through measuring personal-enacted identity gaps, and intervention could be offered to these students. Further research is needed to substantiate which are the best mechanisms to target, and to develop effective interventions.

In addition, it would be of interest in future work to connect these constructs tested in this study to the dynamic constructivist approach (Hong et al., 2000; Luna et al., 2008). Although the present study focuses on the *outcomes* of acculturation, rather than the process of internalizing a new culture, the ways in which an individual switches between different cultural identities during acculturation impacts mental health and well-being. Personal-enacted identity gaps may be a product of frame switching such that the gap between the identity reflected to members of the host culture and the identity perceived by the individual him or herself may be caused by the cultural constructs that guide cognition in different communication settings. For example, a Chinese international student may feel like she is not able to communicate in accordance with her self-view when talking to an American student because the interaction invokes cultural constructs that she is unaccustomed to using and that are not yet fully a part of her identity. Previous research has explored the process of personal-enacted identity gaps by looking at the effects of language and cultural differences, but not frame switching and dynamic constructivist approach. Future studies should investigate this relationship through methods of cultural priming.

## Conclusion

The results of our study highlight the importance of studying personal-enacted identity gaps in order to understand the acculturation challenges for international students. Experiencing depression symptoms is common for international students in the United States; understanding the ways in which personal-enacted identity gaps work through acculturative stress and affect the emotional experiences of international students has important implications for the well-being and mental health of this large portion of the United States student population. University international student events and support groups can potentially contribute to reducing depression symptom levels by directly addressing personal-enacted identity gaps.

## Data Availability Statement

The datasets generated for this study are available on request to the corresponding authors.

## Ethics Statement

This study was carried out in accordance with the recommendations of the Common Rule, Department of Health and Human Services (HHS) through the Office for Human Research Protections (OHRP). The protocol was approved by the Brandeis University Institutional Review Board. All subjects gave written informed consent in accordance with the Declaration of Helsinki.

## Author Contributions

SA led the conception and design of the study and organized the data. SA and HS performed the statistical analyses. SA wrote the first draft of the manuscript. AG drafted the results and edited the manuscript, with HS guiding the writing of the results section and contributing to additional edits. All authors worked to address reviewer comments and read and approved the submitted version.

## Conflict of Interest

The authors declare that the research was conducted in the absence of any commercial or financial relationships that could be construed as a potential conflict of interest.

## References

[B1] Allen M. W. Amason P. Holmes S. (1998). Social support, Hispanic emotional acculturative stress and gender. *Commun. Stud.* 49 139–157. 10.1080/10510979809368525

[B2] Berry J. W. (1970). Marginality, stress and ethnic identification in an acculturated Aboriginal community. *J. Cross-Cult. Psychol.* 1 239–252. 10.1177/135910457000100303

[B3] Berry J. W. (1990). “Acculturation and adaptation: a general framework,” in Mental Health of Immigrants and Refugees. eds Holtzman W. H. Bornemann T. H. (Austin, TX: Hogg Foundation for Mental Health), 90–102. 10.1177/135910457000100303

[B4] Berry J. W. (2006). “Acculturative stress,” in *Handbook of Multicultural Perspectives on Stress and Coping*, eds Holtzman W. H. Bornemann T. H. (Austin, TX: Hogg Foundation for Mental Health), 287–298. 10.1007/0-387-26238-5_12

[B5] Chen J. A. Liu L. Zhao X. Yeung A. S. (2015). Chinese international students: an emerging mental health crisis. *J. Am. Acad. Child & Adolesc. Psychiatry* 54 879–880. 10.1016/j.jaac.2015.06.022 26506576

[B6] Chiao J. Y. Harada T. Komeda H. Li Z. Mano Y. Saito D. (2010). Dynamic cultural influences on neural representations on the self. *J. Cognit. Neurosci.* 22 1–11. 10.1162/jocn.2009.21192 19199421

[B7] Crockett L. J. Iturbide M. I. Torres Stone R. A. McGinley M. Raffaelli M. Carlo G. (2007). Acculturative stress, social support, and coping: relations to psychological adjustment among Mexican American college students. *Cult. Divers. Ethnic Minority Psychol.* 13:347. 10.1037/1099-9809.13.4.347 17967103

[B8] Cross S. E. (1995). Self-construals, coping, and stress in cross-cultural adaptation. *J. Cross-Cult. Psychol.* 26 673–697. 10.1177/002202219502600610

[B9] Hamamura T. Laird P. G. (2014). The effect of perfectionism and acculturative stress on levels of depression experienced by East Asian international students. *J. Multicult. Counsel. Dev.* 42 205–217. 10.1002/j.2161-1912.2014.00055.x

[B10] Han X. Han X. Luo Q. Jacobs S. Jean-Baptiste M. (2013). Report of a mental health survey among Chinese international students at Yale University. *J. Am. Coll. Health* 61 1–8. 10.1080/07448481.2012.738267 23305539

[B11] Hecht M. L. (1993). 2002-a research odyssey: toward the development of a communication theory of identity. *Commun. Monogr.* 60 76–82. 10.1080/03637759309376297

[B12] Henrich J. Heine S. J. Norenzayan A. (2010). The weirdest people in the world? *Behav. Brain Sci.* 33 61–83. 10.1017/s0140525x0999152x 20550733

[B13] Higgins T. (1987). Self-discrepancy: a theory relating self and affect. *Psychol. Rev.* 94 319–340. 10.1037/0033-295x.94.3.319 3615707

[B14] Hong Y. Y. Morris M. W. Chiu C. Y. Benet-Martinez V. (2000). Multicultural minds: a dynamic constructivist approach to culture and cognition. *Am. Psychol*. 55:709. 10.1037/0003-066X.55.7.709 10916861

[B15] Huang S. L. Mussap A. J. (2018). Maladaptive perfectionism, acculturative stress and depression in asian international university students. *J. Psychol. Counsellors Sch.* 28 185–196. 10.1017/jgc.2016.18

[B16] Institute of International Education (2016). “Report on International Educational Exchange,” in *Proceedings of the Open Doors 2016: November 14, 2016* (Washington, DC: National Press Club).

[B17] Jack D. C. (1991). *Silencing the Self: Women and Depression.* Cambridge, MA: Harvard University Press.

[B18] Jackson M. Ray S. Bybell D. (2013). International students in the US: social and psychological adjustment. *J. Int. Stud.* 3 17–28.

[B19] Joiner T. E. Jr. Alfano M. S. Metalsky G. I. (1993). Caught in the crossfire: depression, self-consistency, self-enhancement, and the response of others. *J. Soc. Clin. Psychol.* 12 113–134. 10.1521/jscp.1993.12.2.113

[B20] Jung E. (2013). Delineation of a threefold relationship among communication input variables, identity gaps, and depressive symptoms. *South. Commun. J*. 78, 163–184. 10.1521/jscp.1993.12.2.113

[B21] Jung E. Hecht M. L. (2004). Elaborating the communication theory of identity: identity gaps and communication outcomes. *Commun. Quart.* 52 265–283. 10.1080/01463370409370197

[B22] Jung E. Hecht M. L. (2008). Identity gaps and level of depression among Korean immigrants. *Health Commun.* 23 313–325. 10.1080/10410230802229688 18701996

[B23] Jung E. Hecht M. L. Wadsworth B. C. (2007). The role of identity in international students’ psychological well-being in the United States: a model of depression level, identity gaps, discrimination, and acculturation. *Int. J. Intercult. Relat.* 31 605–624. 10.1016/j.ijintrel.2007.04.001

[B24] Kessler R. C. Petukhova M. Sampson N. A. Zaslavsky A. M. Wittchen H. U. (2012). Twelve-month and lifetime prevalence and lifetime morbid risk of anxiety and mood disorders in the United States. *Int. J. Methods Psychiatr. Res.* 21 169–184. 10.1002/mpr.1359 22865617 PMC4005415

[B25] Kuehner C. (2003). Gender differences in unipolar depression: an update of epidemiological findings and possible explanations. *Acta Psychiatr. Scand.* 108 163–174. 10.1034/j.1600-0447.2003.00204.x 12890270

[B26] Lee A. Y. Aaker J. L. Gardner W. L. (2000). The pleasures and pains of distinct self-construals: the role of interdependence in regulatory focus. *J. Personal. Soc. Psychol.* 78:1122 10.1037//0022-3514.78.6.1122 10870913

[B27] Lee J. Ciftci A. (2014). Asian international students’ socio-cultural adaptation: influence of multicultural personality, assertiveness, academics self-efficacy, and social support. *Int. J. Intercult. Relat.* 38 97–105. 10.1016/j.ijintrel.2013.08.009

[B28] Lee J. S. Koeske G. F. Sales E. (2004). Social support buffering of acculturative stress: a study of mental health symptoms among Korean international students. *Int. J. Intercult. Relat*. 28, 399–414. 10.1016/j.ijintrel.2013.08.009

[B29] Li J. Wang Y. Xiao F. (2019). East Asian international students and psychological well-being: a systematic review. *J. Int. Students* 4, 301–313. 10.1016/j.ijintrel.2013.08.009

[B30] Luna D. Ringberg T. Peracchio L. A. (2008). One individual, two identities: frame switching among biculturals. *J. Consum. Res.* 35 279–293. 10.1086/586914

[B31] Markus H. R. Kitayama S. (1991). Culture and the self: implications for cognition, emotion, and motivation. *Psychol. Rev.* 98:224 10.1037/0033-295x.98.2.224

[B32] Miranda A. O. Matheny K. B. (2000). Socio-psychological predictors of acculturative stress among Latino adults. *J. Mental Health Counsel.* 22:306.

[B33] Mori S. C. (2000). Addressing the mental health concerns of international students. *J. Counsel. Dev.* 78 137–144. 10.1002/j.1556-6676.2000.tb02571.x

[B34] Muthén L. K. Muthén B. O. (2012). *Mplus User’s Guide*, 7th Edn Los Angeles, CA: Muthén & Muthén.

[B35] Nolen-Hoeksema S. (2001). Gender differences in depression. *Curr. Direct. Psychol. Sci.* 10 173–176.

[B36] Oguri M. Gudykunst W. B. (2002). The influence of self construals and communication styles on sojourners’ psychological and sociocultural adjustment. *Int. J. Intercult. Relat.* 26 577–593. 10.1016/s0147-1767(02)00034-2

[B37] Oh Y. Koeske G. F. Sales E. (2002). Acculturation, stress, and depressive symptoms among Korean immigrants in the United States. *J. Soc. Psychol.* 142 511–526. 10.1080/00224540209603915 12153126

[B38] Oyserman D. Lee S. W. (2008). Does culture influence what and how we think? Effects of priming individualism and collectivism. *Psychon. Bull. Rev.* 134 311–342. 10.1037/0033-2909.134.2.311 18298274

[B39] Radloff L. S. (1977). The CES-D scale: a self-report depression scale for research in the general population. *Appl. Psychol. Measure.* 1 385–401. 10.1177/014662167700100306 26918431

[B40] Rice K. G. Choi C. C. Zhang Y. Morero Y. I. Anderson D. (2012). Self-critical perfectionism, acculturative stress, and depression among international students. *Counsel. Psychol.* 40 575–600. 10.1177/0011000011427061

[B41] Sandhu D. S. Asrabadi B. R. (1994). Development of an acculturative stress scale for international students: preliminary findings. *Psychol. Rep.* 75 435–448. 10.2466/pr0.1994.75.1.435 7809315

[B42] Schoemann A. M. Boulton A. J. Short S. D. (2017). Determining power and sample size for simple and complex mediation models. *Soc. Psychol. Personal. Sci.* 8 379–386. 10.1177/1948550617715068

[B43] Singelis T. M. (1994). The measurement of independent and interdependent self-construals. *Personal. Soc. Psychol. Bull.* 20 580–591. 10.1177/0146167294205014

[B44] Smart J. F. Smart D. W. (1995). Acculturative stress of hispanics: loss and challenge. *J. Counsel. Dev.* 73 390–396. 10.1002/j.1556-6676.1995.tb01770.x

[B45] Smith R. A. Khawaja N. G. (2011). A review of the acculturation experiences of international students. *Int. J. Intercult. Relat.* 35 699–713. 10.1016/j.ijintrel.2011.08.004

[B46] Taliaferro L. A. Muehlenkamp J. J. Jeevanba S. B. (2019). Factors associated with emotional distress and suicide ideation among international college students. *J. Am. Coll. Health* 1–5. 10.1080/07448481.2019.1583655 30908153

[B47] Urban E. L. Orbe M. P. (2010). Identity gaps of contemporary US immigrants: acknowledging divergent communicative experiences. *Commun. Stud.* 61 304–320. 10.1080/10510971003757147

[B48] Wadsworth B. C. Hecht M. L. Jung E. (2008). The role of identity gaps, discrimination, and acculturation in international students educational satisfaction in American classrooms. *Commun. Educ*. 57, 64–87. 10.1080/10510971003757147

[B49] Wang W. H. (2009). *Chinese International Students’ Cross-Cultural Adjustment in the US: The Roles of Acculturation Strategies, Self-Construals, Perceived Cultural Distance, and English Self-confidence.* Austin: The University of Texas at Austin.

[B50] Wang Y. Xiao F. (2014). East Asian international students and psychological well-being: a systematic review. *J. Int. Stud.* 4:301.

[B51] Wei M. Heppner P. P. Mallen M. J. Ku T. Y. Liao K. Y. H. Wu T. F. (2007). Acculturative stress, perfectionism, years in the United States, and depression among Chinese international students. *J. Counsel. Psychol.* 54:385 10.1037/0022-0167.54.4.385

[B52] Weinstock L. M. Whisman M. A. (2007). Rumination and excessive reassurance-seeking in depression: a cognitive–interpersonal integration. *Cognit. Ther. Res.* 31 333–342. 10.1007/s10608-006-9004-2

[B53] Williams C. L. Berry J. W. (1991). Primary prevention of acculturative stress among refugees: application of psychological theory and practice. *Am. Psychol.* 46:632 10.1037//0003-066x.46.6.632 1952422

[B54] Yang R. P. J. Noels K. A. Saumure K. D. (2006). Multiple routes to cross-cultural adaptation for international students: mapping the paths between self-construals, English language confidence, and adjustment. *Int. J. Intercult. Relat.* 30 487–506. 10.1016/j.ijintrel.2005.11.010

[B55] Yeh C. J. Inose M. (2003). International students’ reported English fluency, social support satisfaction, and social connectedness as predictors of acculturative stress. *Counsell. Psychol. Quart.* 16 15–28. 10.1080/0951507031000114058

[B56] Zhang J. Goodson P. (2011). Predictors of international students’ psychosocial adjustment to life in the United States: a systematic review. *Int. J. Intercult. Relat.* 35 139–162. 10.1016/j.ijintrel.2010.11.011

