# Supplementary material for: Mind the Gap: The Relation Between Identity Gaps and Depression Symptoms in Cultural Adaptation
Source: Front Psychol. 2020 Jun 5;11:1156. doi: 10.3389/fpsyg.2020.01156 (PMC7289985; doi:10.3389/fpsyg.2020.01156)
Supplement: Supplementary file 1 [file Data_Sheet_1.docx]

Supplementary Table 1

Enrollment breakdown based on region

| **Continent** | **Percentage** |
| --- | --- |
| Europe | 7.6% |
| Central and South America | 5.8% |
| East Asia | 73.7% |
| West Asia | 10.5% |
| Africa | 2.3% |

Supplementary Table 2

Enrollment breakdown based on university

| **University Name** | **Number Enrolled** |
| --- | --- |
| Brandeis University | 69% (118) |
| Columbia University | 8.1%(14) |
| Northeastern University | 9.9% (17) |
| The American Film Institute Barnard University, Baruch College, Berklee College of Music, Boston University, Brown University, Cambridge College, Franklin and Marshall College, New York University, Southern New Hampshire University, Stanford University, Tufts University, University of Alabama System, University of California, University of Illinois, University of Maryland, Baltimore County, University of Pennsylvania, Worcester Polytechnic Institute, Carnegie Mellon University | <5% |

Supplementary Figure 1. Reversed model

Personal-enacted Identity Gap

(Y)

Acculturative Stress

(M)

Depression Symptoms

(X)

Supplementary Table 3

Results for the reversed mediation model: depressive symptoms (X) and personal-enacted identity gaps (Y) mediated by acculturative stress (M).

| Outcome Variable | | Predictor | *b* | SE | *b/SE* | *β* | *p* | *R^2^* |
| --- | --- | --- | --- | --- | --- | --- | --- | --- |
| ACS | | Dep | 0.900 | 0.188 | 4.799 | 0.386 | <.001* | .160 |
|  |  | Age | 0.422 | 0.465 | 0.906 | 0.072 | .365 |  |
|  |  | Gender | 2.578 | 3.590 | 0.718 | 0.115 | .473 |  |
| PEI | | Dep | 0.312 | 0.074 | 4.237 | 0.273 | <.001* | .179 |
|  |  | ACS | 0.111 | 0.039 | 2.820 | 0.226 | .005* |  |
|  |  | Age | -0.372 | 0.214 | -1.739 | -0.129 | .082 |  |
|  |  | Gender | -0.277 | 1.549 | -0.179 | -0.025 | .858 |  |
|  | Indirect Effects | | | | | | |  |
| Dep - PEI Total | | | 0.412 | 0.074 | 5.578 | 0.361 | <.001* |  |
| Dep - PEI Indirect Effect | | | 0.100 | 0.041 | 2.418 | 0.087 | .016* |  |
| Direct Effect of Dep | | | 0.312 | 0.074 | 4.237 | 0.273 | <.001* |  |

* *p<.05*

Supplementary Figure 2. Rotation 1 model

Acculturative Stress

(Y)

Depression Symptoms

(M)

Personal-enacted Identity Gap

(X)

Supplementary Table 4

Results for the rotation 1 mediation model: personal-enacted identity gaps (X), acculturative stress (Y) mediated by depressive symptoms (M).

| Outcome Variable | | Predictor | *b* | SE | *b/SE* | *β* | *p* | *R^2^* |
| --- | --- | --- | --- | --- | --- | --- | --- | --- |
| Dep | | PEI | 0.317 | 0.061 | 5.160 | 0.363 | <.001* | .139 |
|  |  | Age | 0.291 | 0.170 | 1.716 | 0.115 | .086 |  |
|  |  | Gender | -0.467 | 1.539 | -0.304 | -0.048 | .762 |  |
| ACS | | Dep | 0.714 | 0.200 | 3.574 | 0.306 | <.001* | .207 |
|  |  | PEI | 0.461 | 0.153 | 3.013 | 0.227 | .003* |  |
|  |  | Age | 0.571 | 0.440 | 1.297 | 0.097 | .195 |  |
|  |  | Gender | 2.711 | 3.533 | 0.767 | 0.120 | .443 |  |
|  | Indirect Effects | | | | | | |  |
| PEI – ACS Total | | | 0.688 | 0.152 | 4.537 | 0.338 | <.001* |  |
| PEI – ACS Indirect Effect | | | 0.226 | 0.075 | 3.015 | 0.111 | .003* |  |
| Direct Effect of PEI | | | 0.461 | 0.153 | 3.013 | 0.227 | .003* |  |

* *p<.05*

Supplementary Figure 3. Rotation 2 model

Personal-enacted Identity Gap

(Y)

Depression Symptoms

(M)

Acculturative Stress

(X)

Supplementary Table 5

Results for the rotation 2 mediation model: acculturative stress (X), personal-enacted identity gaps (Y), mediated by depressive symptoms (M).

| Outcome Variable | | Predictor | *b* | SE | *b/SE* | *β* | *p* | *R^2^* |
| --- | --- | --- | --- | --- | --- | --- | --- | --- |
| Dep | | ACS | 0.167 | 0.031 | 5.351 | 0.390 | <.001* | .159 |
|  |  | Age | 0.113 | 0.169 | 0.668 | 0.045 | .504 |  |
|  |  | Gender | -0.878 | 1.578 | -0.556 | -0.091 | .578 |  |
| PEI | | Dep | 0.313 | 0.074 | 4.242 | 0.273 | <.001* | .184 |
|  |  | ACS | 0.115 | 0.039 | 2.960 | 0.233 | .003* |  |
|  |  | Age | -0.375 | 0.213 | -1.761 | -0.129 | .078 |  |
|  |  | Gender | -0.179 | 1.548 | -0.116 | -0.016 | .908 |  |
|  | Indirect Effects | | | | | | |  |
| ACS – PEI Total | | | 0.167 | 0.039 | 4.339 | 0.340 | <.001* |  |
| ACS – PEI Indirect Effect | | | 0.052 | 0.016 | 3.233 | 0.107 | .001* |  |
| Direct Effect of ACS | | | 0.115 | 0.039 | 2.960 | 0.233 | .003* |  |

* *p<.05*

Supplementary Figure 4. Rotation 3 model

Depression Symptoms

(Y)

Personal-enacted Identity Gap

(M)

Acculturative Stress

(X)

Supplementary Table 6

Results for the rotation 3 mediation model: acculturative stress (X), depressive symptoms (Y), mediated by personal-enacted identity gaps (M).

| Outcome Variable | | Predictor | *b* | SE | *b/SE* | *β* | *p* | *R^2^* |
| --- | --- | --- | --- | --- | --- | --- | --- | --- |
| PEI | | ACS | 0.167 | 0.039 | 4.339 | 0.340 | <.001* | .121 |
|  |  | Age | -0.339 | 0.218 | -1.558 | -0.117 | .119 |  |
|  |  | Gender | -0.454 | 1.619 | -0.280 | -0.041 | .779 |  |
| Dep | | PEI | 0.228 | 0.061 | 3.725 | 0.261 | <.001* | .219 |
|  |  | ACS | 0.129 | 0.033 | 3.928 | 0.301 | <.001* |  |
|  |  | Age | 0.190 | 0.165 | 1.153 | 0.075 | .249 |  |
|  |  | Gender | -0.774 | 1.509 | -0.513 | -0.080 | .608 |  |
|  | Indirect Effects | | | | | | |  |
| ACS – Dep Total | | | 0.167 | 0.031 | 5.351 | 0.390 | <.001* |  |
| ACS – Dep Indirect Effect | | | 0.038 | 0.014 | 2.738 | 0.089 | .006* |  |
| Direct Effect of ACS | | | 0.129 | 0.033 | 3.928 | 0.301 | <.001* |  |

* *p<.05*

Supplementary Figure 5. Rotation 4 model

Acculturative Stress

(Y)

Personal-enacted Identity Gap

(M)

Depression Symptoms

(X)

Supplementary Table 7

Results for the rotation 4 mediation model: depressive symptoms (X), acculturative stress (Y), mediated by personal-enacted identity gaps (M).

| Outcome Variable | | Predictor | *b* | SE | *b/SE* | *β* | *p* | *R^2^* |
| --- | --- | --- | --- | --- | --- | --- | --- | --- |
| PEI | | Dep | 0.412 | 0.074 | 5.578 | 0.361 | <.001* | .136 |
|  |  | Age | -0.325 | 0.224 | -1.451 | -0.113 | .147 |  |
|  |  | Gender | 0.010 | 1.559 | 0.007 | 0.001 | .995 |  |
| ACS | | PEI | 0.448 | 0.155 | 2.899 | 0.220 | .004* | .202 |
|  |  | Dep | 0.715 | 0.199 | 3.588 | 0.307 | <.001* |  |
|  |  | Age | 0.568 | 0.439 | 1.293 | 0.097 | .196 |  |
|  |  | Gender | 2.575 | 3.532 | 0.729 | 0.114 | .466 |  |
|  | Indirect Effects | | | | | | |  |
| Dep - ACS Total | | | 0.900 | 0.188 | 4.798 | 0.386 | <.001* |  |
| Dep - ACS Indirect Effect | | | 0.185 | 0.076 | 2.427 | 0.079 | .015* |  |
| Direct Effect of Dep | | | 0.715 | 0.199 | 3.588 | 0.307 | <.001* |  |

* *p<.05*

Supplementary Table 8

Comparison of models to the main model (predicted model presented in the main text) using fit criteria (AIC, BIC). The most negative scores reflect the largest improvement over the main model.

| Model | AIC | BIC | AIC Delta Main | BIC Delta Main |
| --- | --- | --- | --- | --- |
| Main | 2745 | 2780 |  |  |
| Reversed | 2782 | 2817 | 37 | 37 |
| Rotation 1 | 2745 | 2780 | 0 | 0 |
| Rotation 2 | 2505 | 2539 | -240 | -241 |
| Rotation 3 | 2505 | 2539 | -240 | -241 |
| Rotation 4 | 2782 | 2817 | 37 | 37 |

Supplementary Analyses

1.1 Consideration of Additional Factors

We also wanted to assess the potential contributions of additional variables that might account for, or otherwise alter, potential relations amongst the variables of interest. Thus we conducted exploratory analyses that included English fluency, years in the United States, and cultural similarity. English fluency was based on the 5-point self-report measure. Cultural similarity was considered using a metric based on cultural values, as derived in Shulgin, Zinkina, & Korotayev, 2017. It is important to note that none of these variables were considered as the basis for predicted effects a priori, and the sampling and study design were not created to address questions regarding these factors. That is, we did not attempt to sample across varying degrees of cultural similarity or fluency, and for the questions in this study, an assay of cultural similarity based on language and communication norms might be a more appropriate measure than the current metric based on values. As shown in Supplementary Table 9, English fluency significantly predicted acculturative stress, and there was a trend for cultural similarity and English fluency to predict depression symptoms, though this did not reach significance. This finding was in line with previous research on predictors of acculturative stress. A study looking at 319 international students from 62 countries found that English proficiency has a direct negative effect on acculturative stress (Taušová, Bender, Dimitrova, & Vijver, 2019). Other studies demonstrate that lower levels of English proficiency are a predictor of acculturative stress (Dao, Lee, & Chang, 2007; Duru & Poyrazli, 2007; Poyrazli, Kavanaugh, Baker & Al-Timimi, 2004; Sumer, Poyrazli, & Grahame, 2008; Yeh & Inose, 2003; Smith & Khawaja, 2011).

1.2 Comparison of subsample of Asian participants

We also tested whether effects held for a less heterogeneous subsample. Because Asians constituted the largest subsample (n = 123), we re-ran the model on this smaller sample, which included participants from East Asia (e.g., China), South Asia (e.g., India), and Southeast Asia (e.g., Vietnam). The results are shown in Supplementary Table 10. The findings from the model with Asian participants largely converge with those of the full sample: as in the full sample, there was a significant total effect of personal-enacted identity gaps on depression, which was significantly mediated by acculturative stress. Personal-enacted identity gaps predicted acculturative stress and acculturative stress predicted depression symptoms; there was a marginal direct effect of personal-enacted identity gaps on depressive symptoms in the mediation model. The percent of the total effect mediated was 46.15%, which is somewhat higher than in the full sample (28.10%); however, this should be interpreted with caution given the smaller sample size.

Supplementary Table 9

Results for the hypothesized mediation model: personal-enacted identity gaps (X) and depression symptoms (Y) mediated by acculturative stress (M), with additional covariates of cultural similarity, years in the US, and English language fluency.

| Outcome Variable | | Predictor | *b* | SE | *b/SE* | *β* | *p* | *R^2^* |
| --- | --- | --- | --- | --- | --- | --- | --- | --- |
| ACS | | PEI | 0.622 | 0.160 | 3.893 | 0.303 | <.001* | .176 |
|  | | Age | 0.646 | 0.471 | 1.373 | 0.108 | .170 |  |
|  |  | Gender | 0.916 | 3.573 | 0.256 | 0.019 | .798 |  |
|  | | Cultural Similar | -1.144 | 4.201 | -0.272 | -0.026 | .785 |  |
|  | | Yrs in US | 1.105 | 1.390 | 0.795 | 0.059 | .426 |  |
|  | | English fluency | -7.657 | 2.485 | -3.080 | -0.236 | .002* |  |
|  | |  |  |  |  |  |  |  |
| Dep | | PEI | 0.232 | 0.061 | 3.817 | 0.265 | <.001* | .282 |
|  |  | ACS | 0.148 | 0.032 | 4.638 | 0.346 | <.001* |  |
|  |  | Age | 0.187 | 0.187 | 1.000 | 0.073 | .317 |  |
|  |  | Gender  Cultural Similar  Yrs in US  English fluency | -0.246  -2.899  -0.756  1.841 | 1.455  1.514  0.532  1.033 | -0.169  -1.915  -1.420  1.783 | -0.012  -0.155  -0.095  0.133 | .866  .055  .155  .075 |  |
|  | Indirect Effects | | | | | | |  |
| PEI – Dep Total | | | 0.324 | 0.063 | 5.136 | 0.370 | <.001* |  |
| PEI – Dep Indirect Effect | | | 0.092 | 0.031 | 2.916 | 0.105 | .004* |  |
| Direct Effect of PEI | | | 0.232 | 0.061 | 3.817 | 0.265 | <.001* |  |

Supplementary Table 10

Results for Asian subsample for the hypothesized mediation model: personal-enacted identity gaps (X) and depression symptoms (Y) mediated by acculturative stress (M).

| Outcome Variable | | Predictor | *b* | SE | *b/SE* | *β* | *p* | *R^2^* |
| --- | --- | --- | --- | --- | --- | --- | --- | --- |
| **Total effects model:** | | | | | | | | |
| **Dep** | | PEI | 0.245 | 0.076 | 3.211 | 0.280 | .001* |  |
|  | | Age | 0.086 | 0.266 | 0.323 | 0.030 | .747 |  |
|  | | Gender | -0.397 | 1.885 | -0.211 | -0.042 | .833 |  |
| **Mediation model:** | | | | | | | | |
| ACS | | PEI | 0.707 | 0.179 | 3.940 | 0.364 | <.001* | .132 |
|  | | Age | -0.064 | 0.703 | -0.091 | -0.010 | .928 |  |
|  |  | Gender | -2.309 | 4.051 | -0.570 | -0.110 | .569 |  |
| Dep | | PEI | 0.134 | 0.072 | 1.862 | 0.154 | .063 | .193 |
|  |  | ACS | 0.162 | 0.037 | 4.336 | 0.361 | <.001* |  |
|  |  | Age | 0.101 | 0.218 | 0.463 | 0.035 | .644 |  |
|  |  | Gender | 0.016 | 1.810 | 0.009 | 0.002 | .993 |  |
|  | Indirect Effects | | | | | | |  |
| PEI – Dep Total | | | 0.249 | 0.076 | 3.251 | 0.286 | .001* |  |
| PEI – Dep Indirect Effect | | | 0.114 | 0.041 | 2.782 | 0.132 | .005* |  |
| Direct Effect of PEI | | | 0.134 | 0.072 | 1.862 | 0.154 | .063 |  |

**References:**

Dao, T. K., Lee, D., & Chang, H. L. (2007). Acculturation level, perceived English fluency, perceived social support level, and depression among Taiwanese international students. College Student Journal, 41(2).

Duru, E., & Poyrazli, S. (2007). Personality dimensions, psychosocial-demographic variables, and English language competency in predicting level of acculturative stress among Turkish international students. International Journal of Stress Management, 14(1), 99.

Hsieh, C.-H. (2000). Self-construals, coping, and the culture fit hypothesis: A cross-cultural study. *Dissertation Abstracts International: Section B: The Sciences and Engineering, 61*(1-B), 588.

Hu, X., Wang, Y., Pruessner, J. C., & Yang, J. (2018). Interdependent self-construal, social evaluative threat and subjective, cardiovascular and neuroendocrine stress response in Chinese. *Hormones and Behavior, 106*, 112-121.

Nolen-Hoeksema, S. (1991). Responses to depression and their effects on the duration of depressive episodes. Journal of abnormal psychology, 100(4), 569.

Poyrazli, S., Kavanaugh, P. R., Baker, A., & Al‐Timimi, N. (2004). Social support and demographic correlates of acculturative stress in international students. Journal of College Counseling, 7(1), 73-82.

Shulgin, S., Zinkina, J., & Korotayev, A. (2017). “Neighbors in values”: A new dataset of cultural distances between countries based on individuals’ values, and its application to the study of global trade. *Research in International Business and Finance, 42*, 966-985.

Smith, R. A., & Khawaja, N. G. (2011). A review of the acculturation experiences of international students. International Journal of Intercultural Relations, 35(6), 699-713.

Sümer, S., Poyrazli, S., & Grahame, K. (2008). Predictors of depression and anxiety among international students. Journal of Counseling & Development, 86(4), 429-437.

Taušová, J., Bender, M., Dimitrova, R., & van de Vijver, F. (2019). The role of perceived cultural distance, personal growth initiative, language proficiencies, and tridimensional acculturation orientations for psychological adjustment among international students. International Journal of Intercultural Relations, 69, 11-23.

Yeh, C. J., & Inose, M. (2003). International students' reported English fluency, social support satisfaction, and social connectedness as predictors of acculturative stress. *Counselling Psychology Quarterly*, *16*(1), 15-28.
